# Childhood Lymphohematopoietic Cancer Incidence and Hazardous Air Pollutants in Southeast Texas, 1995–2004

**DOI:** 10.1289/ehp.11593

**Published:** 2008-08-25

**Authors:** Kristina W. Whitworth, Elaine Symanski, Ann L. Coker

**Affiliations:** 1 Division of Epidemiology and Disease Control, University of Texas School of Public Health, Houston, Texas, USA; 2 Department of Obstetrics and Gynecology, University of Kentucky, Lexington, Kentucky, USA

**Keywords:** 1,3-butadiene, air toxics, benzene, childhood cancer, epidemiology, hazardous air pollution

## Abstract

**Background:**

Cancer is the second leading cause of death among U.S. children with few known risk factors. There is increasing interest in the role of air pollutants, including benzene and 1,3-butadiene, in the etiology of childhood cancers.

**Objective:**

Our goal was to assess whether census tracts with the highest benzene or 1,3-butadiene ambient air levels have increased childhood lymphohematopoietic cancer incidence.

**Methods:**

Our ecologic analysis included 977 cases of childhood lymphohematopoietic cancer diagnosed from 1995–2004. We obtained the U.S. Environmental Protection Agency’s 1999 modeled estimates of benzene and 1,3-butadiene for 886 census tracts surrounding Houston, Texas. We ran Poisson regression models by pollutant to explore the associations between pollutant levels and census-tract cancer rates. We adjusted models for age, sex, race/ethnicity, and community-level socioeconomic status (cSES).

**Results:**

Census tracts with the highest benzene levels had elevated rates of all leukemia [rate ratio (RR) = 1.37; 95% confidence interval (CI), 1.05, 1.78]. This association was higher for acute myeloid leukemia (AML) (RR = 2.02; 95% CI, 1.03–3.96) than for acute lymphocytic leukemia (ALL) (RR = 1.24; 95% CI, 0.92–1.66). Among census tracts with the highest 1,3-butadiene levels, we observed RRs of 1.40 (95% CI, 1.07–1.81), 1.68 (95% CI, 0.84–3.35), and 1.32 (95% CI, 0.98–1.77) for all leukemia, AML, and ALL, respectively. We detected no associations between benzene or 1,3-butadiene levels and lymphoma incidence. Results that examined joint exposure to benzene and 1,3-butadiene were similar to those that examined each pollutant separately.

**Conclusions:**

Our ecologic analysis suggests an association between childhood leukemia and hazardous air pollution; further research using more sophisticated methodology is warranted.

Not only is childhood cancer the second leading cause of death among children in the United States (Jemal et al. 2008), but the etiology of these cancers remains poorly understood (Linabery and Ross 2008), with few established risk factors (Belson et al. 2007; Raaschou-Nielsen and Reynolds 2006). It has been hypothesized that as many as 90% of childhood leukemias, the most common form of childhood cancer, have an unknown etiology (Buffler et al. 2005). Recently, there has been increasing focus on the potential role of environmental toxicants in the development of childhood cancer, including leukemia. Because children are rapidly developing and may metabolize toxicants differently than adults, it is assumed that they are more sensitive to environmental exposures (Goldman 1995; Landrigan et al. 2004; Whyatt and Perera 1995). A recent meta-analysis of studies examining children’s environmental exposures and biomarkers of genetic damage provides evidence of enhanced susceptibility during this critical stage of development (Neri et al. 2006). One environmental exposure receiving increased attention as it relates to maternal and child health, particularly in urban areas, is hazardous air pollution.

Hazardous air pollutants (HAPs) are a class of 188 compounds, including benzene and 1,3-butadiene, which are known or suspected to have adverse effects on health [U.S. Environmental Protection Agency (EPA) 2008]. Benzene is one of the best understood human carcinogens, and 1,3-butadiene is a suspected human carcinogen; much of the evidence regarding their carcinogenicity, however, comes from animal models and studies of adults exposed occupationally (International Agency for Research on Cancer 1982, 1987, 1998). Moreover, a recent investigation demonstrated that Chinese workers exposed to benzene levels ≤ 1 ppm had significantly decreased white blood cell and platelet counts compared with unexposed workers (Lan et al. 2004), suggesting that benzene exposure occurring at levels comparable with what might be expected of ambient air concentrations has the potential to adversely affect health.

Although the general population is exposed to background levels of benzene and 1,3-butadiene, those living in urban areas with increased traffic density or in close proximity to chemical manufacturing facilities or petroleum refineries are likely exposed to even higher levels of these compounds (Agency for Toxic Substance and Disease Registry 1992, 2007). Despite ubiquitous levels of benzene and 1,3-butadiene in the ambient air environment, there have been few studies of cancer risk among children living in urban communities with potentially high levels of HAPs. Most of these studies have used proxy measures of exposure to traffic-related air pollution, and results have been mixed (Crosignani et al. 2004; Harrison et al. 1999; Knox 2006; Nordlinder and Jarvholm 1997; Pearson et al. 2000; Raaschou-Nielsen et al. 2001; Reynolds et al. 2002; Steffen et al. 2004). Two other studies have reported increased risk of childhood cancers among children whose residence was near a “hot spot” of benzene or 1,3-butadiene emissions (Knox 2005a, 2005b). Reynolds et al. (2003) found 21% [rate ratio (RR) = 1.21; 95% confidence interval (CI), 1.03–1.42] higher rates of childhood leukemia among census tracts with the highest HAP exposure score (for 25 individual pollutants combined) compared with census tracts with the lowest scores. It has been suggested that the inconsistencies in the current air pollution and childhood cancer literature may be attributable to differences in analytic and exposure assessment methodology as well as differences in the sampled populations’ underlying genetic susceptibility (Buffler et al. 2005).

Given the limitations of the current literature regarding the potential association between childhood cancer incidence and ambient HAPs, we conducted an ecologic study in southeastern Texas to determine whether census tracts with the highest estimated levels of benzene and 1,3-butadiene have higher incidence rates of childhood lymphohematopoietic cancer compared with census tracts with the lowest estimated levels. Central to this study area is Harris County, home of the Houston Ship Channel and a vast number of petroleum and chemical industries operating in its borders (Sexton et al. 2007). Additionally, the study area includes the city of Houston, a large metropolitan area covered by a dense network of roadways. Given the presence of both point and mobile sources of exposure to benzene and 1,3-butadiene, Harris and surrounding counties provided an ideal location to further study potential childhood cancer risks associated with levels of benzene and 1,3-butadiene in ambient air.

## Materials and Methods

### Study population

We identified 997 cases of lymphohematopoietic cancer among children < 20 years of age from the Texas Cancer Registry (TCR), a North American Association of Central Cancer Registries (2007) gold-certified population-based registry. All cases were diagnosed between 1995 and 2004 and resided in one of the following eight counties surrounding Houston, Texas, at the time of diagnosis: Harris, Montgomery, Liberty, Chambers, Fort Bend, Brazoria, Waller, and Galveston. The specific lymphohematopoietic cancers identified were leukemia [*International Classification of Diseases, 10th Revision* (ICD-10) (World Health Organization 1993) codes C91–C95; *n* = 702], non-Hodgkin’s lymphoma (NHL) (ICD-10 codes C82–C85; *n* = 140), and Hodgkin’s disease (ICD-10 code C81; *n* = 155). We additionally examined, in separate analyses, the two most common types of childhood leukemia: acute lymphocytic leukemia (ALL) and acute myeloid leukemia (AML). We identified 531 cases of ALL and 99 cases of AML. The study protocol was reviewed and approved by the Texas Department of State Health Services and the University of Texas Health Science Center Committee for the Protection of Human Subjects.

### Exposure assessment

We based the exposure assessment on the address at which the case resided at the time of diagnosis, as reported by the TCR. We excluded 11 cases because their reported address was that of a hospital or other known medical facility. The TCR reported the exact latitude and longitude of the reported residence for 874 (89%) of the remaining cases. For the other 112 cases, the TCR either did not provide the latitude and longitude or provided coordinates for the ZIP code centroid. For these addresses, where applicable, we removed apartment numbers and unnecessary spaces, spelled out abbreviations for farm-to-market road, state highway, and county road, and double-checked the spelling of street names. Once we made these modifications of the addresses, we used AtlasGIS software (version 4.0; ESRI, Redlands, CA) to successfully geocode 47 addresses. The remaining addresses (*n* = 65) were largely post office boxes, rural routes, or incomplete addresses (addresses with only a partial street name or missing a house number). We “matched” these cases to another case based on reported ZIP code, race/ethnicity, and age group and assigned it to the same census tract as their “match.” Only nine cases (< 1% of the total) remained for whom an appropriate “match” could not be found, and were therefore excluded.

We obtained population estimates, stratified by race/ethnicity (non-Hispanic white, non-Hispanic black, Hispanic, and other), sex, and age group (0–4, 5–9, 10–14, and 15–19 years), for each of the 886 census tracts in our eight-county study area from the 2000 U.S. Census Bureau (U.S. Census Bureau 2006). We further excluded 24 cases from analyses because the 2000 census estimated a zero population total for their strata (defined by race/ethnicity, sex, and age group) in the census tract. This left 670 cases of leukemia (510 cases of ALL and 92 cases of AML), 146 Hodgkin’s disease cases, and 137 cases of NHL eligible for analysis.

We obtained estimates of ambient air levels of benzene and 1,3-butadiene from the U.S. EPA 1999 National-Scale Air Toxics Assessment (NATA) project. The goal of NATA was to characterize population risk from 177 HAPs in ambient air, including benzene and 1,3-butadiene (U.S. EPA 2008). As part of this project, the U.S. EPA used a computer simulation model, the Assessment System for Population Exposure Nationwide (ASPEN), to estimate annual levels of HAPs for every census tract in the contiguous United States (U.S. EPA 2007). The ASPEN model is based on emissions data for the year in which estimates are made (1999 in our case), and it also accounts for many other factors known to influence ambient air levels of HAPs. These factors include meteorologic data (wind speed and direction), the rate and height of the release, and reactive decay, as well as deposition and secondary formation (U.S. EPA 2007). The census-tract–level ASPEN data for benzene and 1,3-butadiene also include an estimate of background levels of these pollutants, based on available monitoring data (U.S. EPA 2007). Although the ASPEN model produces separate estimates for each type of major emission source [mobile, area, and major (point)], our assessment relied upon the modeled ambient levels of benzene and 1,3-butadiene from all sources combined. We categorized census tract estimates of air pollutant levels into quartiles based on the distribution in the study area and treated them as a nominal variable in all analyses.

### Covariates

We treated age at diagnosis, sex, race/ethnicity, and community-level socioeconomic status (cSES) as confounders. We assessed age at diagnosis as a categorical variable with four levels (0–4, 5–9, 10–14, and 15–19 years of age). We categorized race/ethnicity as non-Hispanic white, non-Hispanic black, Hispanic, and other. Rather than excluding cases with an unknown race/ethnicity (*n* = 6), we included them in the “other” category because we felt the potential loss of power was less desirable than the minimal effects resulting from the addition of these cases to an already heterogeneous group.

“Socioeconomic status” is a complex term and involves such attributes as income, education level, and household characteristics (Greenwald et al. 1996). Because the TCR does not compile information on measures of these attributes in its database, we constructed a composite index of socioeconomic status based on census-tract–level data from the 2000 Census. We used Stata (version 8; StataCorp, College Station, TX) to conduct a principal components analysis to identify the most relevant factors. The census-tract–level factors we evaluated for inclusion in the composite variable were median household income, median house value, median rent, percent high school diploma, percent college diploma, percent professional degree, percent employed, and percent below the poverty line. The factors included in the final cSES variable, based on their factor loadings, were percent high school diploma, percent professional degree, median household income, median house value, median rent, and percent below poverty. We categorized this variable into quartiles based on its distribution in the entire state of Texas and treated it as an ordinal variable in analyses.

### The association between HAPs and lymphohematopoietic cancer incidence

We used Poisson regression analyses to explore the association between census-tract–level estimates of benzene and 1,3-butadiene in ambient air and lymphohematopoietic cancer incidence. We conducted analyses separately for each of the following lymphohematopoietic cancer subtypes: all leukemia, ALL, AML, Hodgkin’s disease, and NHL. The dependent variable was cancer case count, indexed by census tract, sex, race/ethnicity, and age group. Air pollutant level was the primary exposure under investigation, and we initially ran models separately for each pollutant. Other independent variables included in the model were sex, age group, race/ethnicity, and cSES. We used an offset equal to the log of the 9-year population total for each census tract, sex, and age group combination to account for the different population sizes in each of these groups. We used generalized estimating equations to account for clustering of incidence rates within census tracts. To test for trend, we ran separate models treating air pollutant level in quartiles as an ordinal variable, and we report the Wald *p*-values for trend.

Because preliminary analyses indicated that benzene and 1,3-butadiene levels (broken down by quartiles) were highly correlated (Spearman correlation coefficient = 0.91, *p* < 0.0001), we were unable to include these two variables in the same regression model because of issues associated with collinearity. However, in an effort to consider their joint effect, we created another variable by classifying census tracts based on our quartile categorization of the ASPEN modeled estimates for benzene and 1,3-butadiene. For this “joint” exposure variable, we ranked census tracts in the highest quartile for both 1,3-butadiene and benzene levels as “high,” and those in the lowest quartile as “low,” which served as the referent category. We assigned all other census tracts to the middle category. Using this joint exposure variable, we reran analyses for all cancer subtypes. We completed all analyses in SAS (version 9.1; SAS Institute Inc., Cary, NC).

## Results

Table 1 presents the number of cases of each type of lymphohematopoietic cancer included in our study by age, sex, race/ethnicity, and cSES. Cases of ALL and AML were diagnosed at younger ages, whereas Hodgkin’s disease cases were generally diagnosed at older ages. Overall, there were a higher proportion of male than female cases. A greater proportion of ALL and AML cases were Hispanic, whereas most Hodgkin’s disease and NHL cases were non-Hispanic white. Additionally, regardless of cancer type, our population had a higher proportion of cases in the highest quartile of cSES.

Table 2 presents the distribution of the U.S. EPA ASPEN model estimates for benzene and 1,3-butadiene in the study area. Across the 886 census tracts in our study area, ambient air levels ranged from 0.42 to 9.05 μg/m^3^ for benzene and from 0.01 to 2.41 μg/m^3^ for 1,3-butadiene. The median benzene level in the eight-county study area (1.72 μg/m^3^) is approximately 10 times the median level of 1,3-butadiene (0.16 μg/m^3^).

We found that census tracts with the highest ambient air levels of benzene had elevated rates of all leukemia (RR = 1.37; 95% CI, 1.05–1.78) and AML (RR = 2.02; 95% CI, 1.03–3.96) compared with census tracts with the lowest estimated levels (Table 3). Additionally, these census tracts had 1.24 (95% CI, 0.92–1.66) times the rate of ALL compared with census tracts with the lowest levels, although this estimate was not statistically significant. We detected a statistically significant trend of increasing incidence rates with increasing estimated levels of benzene for all leukemias combined (*p* = 0.03) and a borderline significant trend for AML (*p* = 0.06). We observed no trend for ALL. No associations were observed between benzene levels in ambient air and Hodgkin’s disease or NHL.

Relative to census tracts with the lowest levels, we observed significantly increased rates of all leukemia among census tracts with the highest levels of ambient 1,3-butadiene (RR = 1.40; 95% CI, 1.07–1.81) as well as a significant trend (*p* = 0.01) (Table 4). We also observed 32% (RR = 1.32; 95% CI, 0.98–1.77) and 68% (RR = 1.68; 95% CI, 0.84–3.35) higher (albeit insignificant) rates of ALL and AML, respectively, in these census tracts compared with census tracts with the lowest levels of 1,3-butadiene. We found a borderline significant trend between rates of ALL (*p* = 0.06) and 1,3-butadiene levels. We observed no statistically significant trend between rates of AML and 1,3-butadiene levels. We detected no association between estimated 1,3-butadiene levels and incidence of Hodgkin’s disease or NHL.

In examining the effect of concomitant exposure to benzene and 1,3-butadiene, for each specific cancer type examined, the estimated RRs comparing the highest exposure group to the lowest were similar in magnitude to the results from the analyses of the independent effects of benzene and 1,3-butadiene (data not shown). However, only the point estimate for all leukemias remained statistically significant.

## Discussion

We found significantly higher rates of leukemia in census tracts with the highest ambient levels of benzene and 1,3-butadiene, as estimated from the U.S. EPA ASPEN model for 1999 (U.S. EPA 2007). We also observed elevated rates of the two most common types of childhood leukemia, ALL and AML, associated with benzene levels. We detected similar results for 1,3-butadiene, although these results were not statistically significant. Our results are consistent with the only other study to have examined childhood cancer rates with ambient air levels of HAPs (Reynolds et al. 2003). Similar to our investigation, Reynolds et al. (2003) used the U.S. EPA ASPEN model for 25 HAPs to create a single exposure score; they did not, however, examine rates in relation to individual pollutants. Reynolds et al. (2003) reported significantly higher rates of childhood leukemia (RR = 1.21; 95% CI, 1.03–1.42) and increased rates of ALL and AML (ALL: RR = 1.19; 95% CI, 1.00–1.43; AML: RR = 1.46; 95% CI, 0.97–2.19) among census tracts with the highest HAP exposure score.

The few other studies that have explored the potential association between childhood cancer and air pollution have produced equivocal results. Of four studies reporting statistically significant positive results, three used measures of traffic density (Crosignani et al. 2004; Nordlinder and Jarvholm 1997; Pearson et al. 2000), and one study examined residential distance from roadways (Knox 2006) to assess exposure. In contrast, two case–control studies found no association between traffic density and childhood leukemia (Reynolds et al. 2002; Steffen et al. 2004), and one case–control study found no significant risk of leukemia associated with residence within 100 m of either a main road or a gas station (Harrison et al. 1999).

Our study has the benefit of being population based and not subject to selection bias. With more than a decade of cancer incidence data for the greater Houston area, we also had sufficient power to evaluate risk associated with HAP exposure. Additionally, we chose to study childhood cancers, which have a shorter latency period than adult cancers and therefore present fewer methodologic challenges when studying environmental exposures. We used an innovative method of assigning cases with “difficult” or “nongeocodable” addresses, most of which were post office boxes, to census tracts by “matching” them to another case with similar demographic characteristics and reporting the same residential ZIP code. This method of assignment is an improvement over discarding data for subjects for whom an exact match cannot be made or by assigning these cases to the ZIP code centroid, which may create an artificial cluster of cases in one location and result in nondifferential exposure misclassification (Hurley et al. 2003).

A limitation of our study is the potential for ecologic fallacy, which may result when using grouped versus individual-level data. Nonetheless, an ecologic study design is efficient when little is known about the association under study (Friis and Sellers 2004), as is the case for childhood cancer and HAPs. Moreover, the smaller the geographic unit of analysis, the more likely bias resulting from aggregation will be minimized (Diggle and Elliott 1995). We chose to analyze data at the census tract level, which we felt to be the smallest possible spatial resolution at which to aggregate without sacrificing so much power that we could no longer detect an association. Because of the aggregate nature of our composite measure of socioeconomic status, there was likely misclassification of this variable. Also, the U.S. Census does not provide intercensus estimates at the sub-county level. Moreover, in our study, it was necessary to have population totals by race/ethnicity, age, and sex for each census tract (32 groups in total), and the accuracy of population projections for small areas has been called into question (Smith and Shahidullah 1995). Given these difficulties, we chose to develop our 10-year subpopulation estimates using the 2000 U.S. Census (U.S. Census Bureau 2006) even though we recognize that there was likely inter-census variability in population growth or decline during this period.

We based our exposure assignment on address at diagnosis, which may not represent the address at which the case or case mother resided during the prenatal or postnatal periods, which may be etiologically more relevant. Few studies have examined the degree of residential stability in epidemiologic investigations (Canfield et al. 2006; Hurley et al. 2003), and we are aware of none that have examined this issue in studies of childhood cancer. To examine possible effects of exposure misclassification due to residential mobility, we ran sensitivity analyses restricting cases to those diagnosed at ages < 5 years. For NHL, which had a majority of cases diagnosed at later ages, we found increased rates compared with the analyses including children of all ages (benzene: RR = 1.49; 95% CI, 2.04–4.54; 1,3-butadiene: RR = 1.61; 95% CI, 0.53–4.90). We also repeated the sensitivity analysis for all leukemias and each subtype (there were too few cases of Hodgkin’s disease diagnosed before 5 years of age to analyze) and found results similar to those presented among all children. These results indicate that mobility is likely not as large a problem for childhood cancers that are diagnosed earlier, such as leukemia, compared with those that are diagnosed at later ages (e.g., NHL).

The ASPEN data provide the advantage of a complex dispersion modeling approach using myriad information about sources and fate and transport of pollutants. The ASPEN model accounts for multiple factors that influence HAP levels in the environment (e.g., meteorologic conditions, emission height, deposition, and secondary formation) and represents a more robust measure of potential exposure than has previously been used in studies of childhood cancer and HAPs. Despite this advantage, our exposure assessment is subject to error. Limitations of the modeling approach include uncertainties in the emissions inventory and the inherent inability to capture variability within a census tract (Ozkaynak et al. 2008). Additionally, we relied upon levels of benzene and 1,3-butadiene in ambient air in 1999. Because cases were diagnosed in 1995–2004, the etiologic window of exposure varied over calendar time, so some misclassification of exposure is likely. On the one hand, data for a single year in the midpoint of this interval may be suitable surrogates for levels over longer periods because the relative ranking of geographic areas of high versus low ambient air pollutant levels (because of their proximity to roadways and point sources) likely remains the same. On the other hand, significant changes in air pollutant emissions due, for example, to the opening or closing of a large industrial facility during the study period could potentially affect the relative ranking of census tracts and result in some misclassification.

Because the ASPEN data represent levels of HAPs in the outdoor environment, data regarding levels in the home or other indoor environments were not available. Because children tend to spend a great deal of time indoors (Adgate et al. 2004), indoor sources of benzene and 1,3-butadiene, such as environmental tobacco smoke, may be a significant contributor to their personal exposure. However, parental smoking has been weakly and inconsistently associated with childhood leukemias in the epidemiologic literature (Belson et al. 2007). Nonetheless, we attempted to control for smoking prevalence by creating a census-tract–level variable using information from county-level rates on smoking by ethnicity, available from the Texas Department of State Health Service’s Behavioral Risk Factor Surveillance System (Texas Department of State Health Services 2005). However, effects associated with air pollutant levels were essentially unchanged with the inclusion of this variable in the model (data not shown), and we chose to report on the results from the more parsimonious model.

Because of the high correlation between estimated ambient air levels of benzene and 1,3-butadiene, we were limited in our ability to tease apart the effects of each pollutant. We attempted to address this issue by creating a single variable representing their joint effect. It is of interest that the comparison of census tracts with high levels of both benzene and 1,3-butadiene levels to census tracts with low levels of both pollutants resulted in RRs similar to, although more imprecise than, those produced by separate analyses of each pollutant. These results suggest that childhood leukemia risk may be related to one, but likely not both, pollutants. Given the overwhelming evidence of the carcinogenic potential of benzene, we therefore cannot exclude the possibility that the associations we observed between childhood cancer and 1,3-butadiene were actually attributable to benzene, and more research is needed to elucidate the cancer risks arising from complex air pollutant mixtures.

## Conclusion

Our exploratory analysis suggests that estimated ambient levels of benzene and 1,3-butadiene may contribute to increased rates of childhood leukemia in census tracts with the highest estimated levels. To our knowledge, this is the first epidemiologic study to have examined childhood cancer incidence rates associated with these two HAPs in Texas. Although data from the U.S. EPA ASPEN model provide a unique source of potential exposure data for researchers interested in the health effects of air pollution, and it is a logical source of data for exploratory analyses, they represent modeled levels rather than actual measured concentrations of HAPs and carry with them some limitations, as discussed above. Given that Houston, Texas, is one of the most densely monitored cities in the nation (Texas Commission on Environmental Quality 2005), work is under way to apply spatial analysis techniques to the existing monitoring data to further explore associations between childhood lymphohematopoietic cancers and hazardous air pollution.

## Figures and Tables

**Table 1:** Number of childhood cancer cases in Harris, Liberty, Montgomery, Chambers, Fort Bend, Waller, Brazoria, and Galveston Counties, Texas, 1995–2004, with geocodable address at diagnosis, by age, sex, race/ethnicity, and cSES [*n* (%)].

Characteristic	All leukemia (*n* = 670)	ALL (*n* = 510)	AML (*n* = 92)	Hodgkin’s disease (*n* = 146)	NHL (*n* = 137)
Age (years)					
< 5	309 (46.1)	244 (47.8)	37 (40.2)	4 (2.7)	28 (20.4)
5–9	146 (21.8)	127 (24.9)	13 (14.1)	12 (8.2)	39 (28.5)
10–14	116 (17.3)	85 (16.7)	15 (16.3)	41 (28.1)	25 (18.3)
15–19	99 (14.8)	54 (10.6)	27 (29.4)	89 (61.0)	45 (32.8)
Sex					
Male	364 (54.3)	274 (53.7)	52 (56.5)	93 (63.7)	97 (70.8)
Female	306 (45.7)	236 (46.3)	40 (43.5)	53 (36.3)	40 (29.2)
Race/ethnicity					
Non-Hispanic white	265 (39.6)	198 (38.8)	36 (39.1)	64 (43.8)	71 (51.8)
Non-Hispanic black	57 (8.5)	38 (7.5)	15 (16.3)	30 (20.6)	15 (11.0)
Hispanic	315 (47.0)	249 (48.8)	37 (40.2)	41 (28.1)	43 (31.4)
Other	33 (4.9)	25 (4.9)	4 (4.4)	11 (7.5)	8 (5.8)
cSES^a^					
1 (low)	142 (21.2)	107 (21.0)	24 (26.1)	30 (20.5)	19 (13.9)
2	139 (20.7)	113 (22.2)	16 (17.4)	29 (19.9)	30 (21.9)
3	152 (22.7)	109 (21.4)	22 (23.9)	32 (21.9)	34 (24.8)
4 (high)	237 (35.4)	181 (35.5)	30 (32.6)	55 (37.7)	54 (39.4)

a Contains information from the following factors: percent high school diploma, percent professional degree, median household income, median house value, median rent, and percent below poverty. We categorized cSES into quartiles based on its distribution in Texas.

**Table 2:** Distribution of 1999 EPA ASPEN modeled estimates of benzene and 1,3-butadiene in ambient air in the eight-county study area (*n* = 886 census tracts).

	Benzene (μg/m^3^)	1,3-Butadiene (μg/m^3^)
Arithmetic mean	1.95	0.19
Geometric mean (95% CI)	1.72 (1.04–2.84)	0.14 (0.07–0.30)
Geometric SD	1.65	2.14
Minimum	0.42	0.01
Maximum	9.05	2.41
Percentile		
25th	1.28	0.11
50th	1.72	0.16
75th	2.36	0.21
90th	3.18	0.31
99th	5.50	0.60

**Table 3:** Adjusted RRs for the association between 1999 U.S. EPA ASPEN modeled estimates of ambient benzene levels and lymphohematopoietic cancer incidence among children (< 20 years of age).

Cancer type	Benzene level	No. of census tracts	No. of cases	Adjusted RR^a^ (95% CI)	*p*-Value	*p*-Value for trend
Hodgkin’s disease	Low	221	36	1.00		
	Medium-low	222	42	1.17 (0.74–1.85)	0.500	
	Medium-high	221	31	1.07 (0.66–1.72)	0.789	
	High	222	37	1.52 (0.92–2.52)	0.101	0.163
NHL	Low	221	42	1.00		
	Medium-low	222	40	0.96 (0.62–1.48)	0.853	
	Medium-high	221	27	0.84 (0.52–1.36)	0.484	
	High	222	28	1.06 (0.60–1.87)	0.852	0.936
All leukemia	Low	221	157	1.00		
	Medium-low	222	183	1.13 (0.91–1.40)	0.269	
	Medium-high	221	156	1.10 (0.87–1.40)	0.415	
	High	222	174	1.37 (1.05–1.78)	0.019	0.034
ALL	Low	221	116	1.00		
	Medium-low	222	147	1.23 (0.97–1.56)	0.092	
	Medium-high	221	123	1.14 (0.87–1.49)	0.349	
	High	222	124	1.24 (0.92–1.66)	0.153	0.186
AML	Low	221	21	1.00		
	Medium-low	222	20	0.93 (0.50–1.72)	0.813	
	Medium-high	221	20	1.11 (0.59–2.10)	0.747	
	High	222	31	2.02 (1.03–3.96)	0.040	0.063

a Adjusted for age, sex, race/ethnicity, and cSES.

**Table 4:** Adjusted RRs for the association between 1999 U.S. EPA ASPEN modeled estimates of ambient 1,3-butadiene levels and lymphohematopoietic cancer incidence among children (< 20 years of age).

Cancer type	1,3-Butadiene level	No. of census tracts	No. of cases	Adjusted RR^a^ (95% CI)	*p*-Value	*p*-Value for trend
Hodgkin’s disease	Low	221	36	1.00		
	Medium-low	221	43	1.16 (0.73–1.82)	0.530	
	Medium-high	221	32	1.10 (0.69–1.76)	0.694	
	High	223	35	1.41 (0.85–2.34)	0.186	0.236
NHL	Low	221	42	1.00		
	Medium-low	221	36	0.85 (0.54–1.34)	0.485	
	Medium-high	221	33	1.01 (0.63–1.61)	0.965	
	High	223	26	0.99 (0.57–1.71)	0.960	0.986
All leukemia	Low	221	147	1.00		
	Medium-low	221	184	1.22 (0.98–1.52)	0.081	
	Medium-high	221	172	1.25 (0.99–1.58)	0.066	
	High	223	167	1.40 (1.07–1.81)	0.013	0.013
ALL	Low	221	108	1.00		
	Medium-low	221	145	1.31 (1.02–1.68)	0.034	
	Medium-high	221	136	1.31 (1.00–1.71)	0.052	
	High	223	121	1.32 (0.98–1.77)	0.064	0.056
AML	Low	221	20	1.00		
	Medium-low	221	23	1.12 (0.61–2.06)	0.721	
	Medium-high	221	22	1.20 (0.64–2.27)	0.570	
	High	223	27	1.68 (0.84–3.35)	0.142	0.163

a Adjusted for age, sex, race/ethnicity, and cSES.
